# Transcription Start Site Choice Regulates m^6^A Stoichiometry in Cap-Proximal Regions

**DOI:** 10.3390/genes17060653

**Published:** 2026-05-31

**Authors:** Jianheng Fox Liu, Samie R. Jaffrey

**Affiliations:** Department of Pharmacology, Weill Cornell Medicine, Cornell University, New York, NY 10065, USA; jil4026@med.cornell.edu

**Keywords:** m^6^A, m^6^Am, transcription start site, RNA modification, GLORI, CROWN-seq, 5′ UTR, cap-proximal region

## Abstract

**Background/Objectives:** *N*6-methyladenosine (m^6^A) is the most prevalent and functionally significant internal modification within eukaryotic mRNA. While m^6^A is known to be regulated at internal sites by factors such as splice junctions, the mechanisms governing deposition within the cap-proximal region remain poorly understood. This study aims to determine the patterns of m^6^A stoichiometry in cap-proximal regions and to investigate whether the choice of the specific transcription start site (TSS) can affect m^6^A stoichiometry. **Methods:** We re-analyzed our published single-nucleotide-resolution CROWN-seq data to quantify m^6^A stoichiometry across transcript isoforms with different TSSs, and assessed the relationship between specific TSSs and specific m^6^A sites (“TSS-m^6^A-site pairs”). **Results:** We established the first single-nucleotide-resolution dataset of m^6^A stoichiometry across the transcriptome in cap proximal regions, including stoichiometry of m^6^A across 5′ isoforms for each gene. We found that m^6^A deposition is markedly inhibited within a narrow cap-proximal region in a distance-sensitive manner. m^6^A sites located close to both 5′ and 3′ exon ends exhibit low methylation due to the overlap between the cap-proximal and 3′ exon-end exclusion zones. **Conclusions:** We find that the first exon contains a narrow m^6^A exclusion zone at its 5′ end. As a result, cap-proximal m^6^A sites can have different stoichiometries depending on the TSS choice. As the m^6^A site is positioned farther from the TSS, m^6^A stoichiometry increases. These results reveal that TSS switching is a regulatory mechanism for m^6^A stoichiometry in cap-proximal regions and provide a mechanism for fine-tuning gene expression and mRNA fate through isoform-specific m^6^A modification stoichiometry.

## 1. Introduction

N6-methyladenosine (m^6^A) is the most prevalent modified nucleotide in mRNA in human cells [1,2]. Previous studies have shown that m^6^A is critical for multiple molecular processes, including RNA stability, splicing, and targeting to stress granules [2,3,4]. Dysfunction of m^6^A deposition is strongly linked to significant pathologies, including various cancers, neurological disorders, and immunological diseases [3,4]. Therefore, there is considerable interest in characterizing the molecular mechanisms that govern how m^6^A is deposited across the transcriptome.

One important question is why m^6^A is deposited unevenly throughout the mRNA transcript. It is known that m^6^A is deposited by the m^6^A writer complex, which preferentially modifies adenosines within the canonical DRACH (D = A, G, or U; R = A or G; H = A, C, or U) motif [5]. However, early studies demonstrated that across mRNA transcripts, DRACH motifs around stop codons are selectively methylated [6]. Recently, an “exon-end exclusion zone” model was proposed to solve this spatial puzzle. This model was suggested by the discovery that m^6^A deposition is repressed around the edges of exons due to competition between the m^6^A writing complex and the splicing machinery, including the exon junction complex (EJC) [2,7,8,9,10]. The splicing machinery comprises a large multiprotein complex that can span over ~100 nt on either side of the exon–exon junction. Since most exons are ~88 to 165 nt (25th to 75th quantile), they are completely covered by the splicing machinery, explaining the low stoichiometry of m^6^A in these exons [8,9,10]. However, for exons >400 nt, the central part of the exon is not covered by the splicing machinery, thus explaining why DRACH sites in the middle of long exons tend to be highly methylated [8,9,10]. Overall, these studies establish that m^6^A stoichiometry is regulated by the exon architecture [11]. Although much is known about m^6^A levels in large internal exons and the role of splicing machinery in shaping their stoichiometry, much less is known about m^6^A stoichiometry in 5′ regions of RNA. This region is derived from the first exon, which lacks an EJC binding on its 5′ side. Thus, it is unclear whether an exclusion zone exists in this region and if m^6^A stoichiometry is related to its position in the 5′ exon.

One major challenge to studying m^6^A sites in the 5′ exon is the lack of m^6^A stoichiometry data. Part of the reason why m^6^A stoichiometry is poorly annotated in mRNA 5′ regions is that current methods for determining m^6^A stoichiometry typically exhibit poor coverage in 5′ regions. Conventional methods, including antibody-based techniques like miCLIP and MeRIP-seq, as well as chemical methods like GLORI, often yield disproportionately low sequencing depth around 5′ ends [5,6,12]. Other methods that also provide quantitative m^6^A stoichiometry measurements, like Oxford Nanopore direct RNA sequencing, have low accuracy in 5′ regions [13]. Lastly, 5′ ends show considerable isoform diversity, and it is possible that m^6^A sites in 5′ regions could be affected by these 5′ isoforms. Indeed, we recently showed that 5′ isoform diversity can be substantial for many genes [14]. Therefore, while multiple studies have reported the importance of the 5′ region m^6^A [15,16,17,18], it remains unclear whether the stoichiometry of these sites is correctly determined or if they possess specific biological functions.

In this study, we describe m^6^A stoichiometries in mRNA 5′ UTRs, and we show that 5′ m^6^A stoichiometry can be highly affected by 5′ UTR isoform diversity. To assess 5′ m^6^A stoichiometry throughout the transcriptome, we used CROWN-seq, a method that uses the chemical conversion method of GLORI to quantify stoichiometries of cap-associated m^6^Am in a 5′ isoform-specific manner [14]. By re-analyzing CROWN-seq data, we established a dataset for m^6^A sites across different 5′ isoforms, termed “TSS-m^6^A-site pairs”. By analyzing these pairs, we found that m^6^A deposition is markedly repressed in the cap-proximal region, specifically within the first 30–40 nucleotides. We also identified joint repression of m^6^A deposition driven by both the proximity to the cap and the 3′ end of the first exon. Our data indicate that an m^6^A site can have highly variable stoichiometry depending on the specific transcription start site (TSS) used in the transcript isoform, revealing an important role for alternative 5′ isoforms in establishing the stoichiometry of nearby m^6^A sites. Overall, this study reveals a novel cap-proximal mechanism for the spatial regulation of m^6^A deposition.

## 2. Materials and Methods

### 2.1. Genomic Assembly and Gene Annotations

Gencode v45 (GRCh38) was used in this study.

### 2.2. Reanalysis of Published Sequencing Data

The GLORI data of HEK293T samples were obtained from the Supplementary Table of the original study [12].

The CROWN-seq data were obtained from our previous study (GSE233655) [14]. Note that the HEK293T samples designated as replicate #1 and #2 are the corresponding samples designated as technical replicates #3 and #4 in our previous study. Replicates #1 and #2 were excluded because their low sequencing depths were insufficient for the analyses.

### 2.3. Estimating CROWN-Seq Non-Conversion Background

We used the RNA spike-in derived from ERCC-00057 to estimate the internal non-conversion background in CROWN-seq. These ERCC spike-ins were made via T7 in vitro transcription with Am/m^6^Am cap-analogs (details see our previous study [14]). The sequences of the RNA spike-ins are: *TAATACGACTCACTATA*AGTGCATCCTCGTGGCATCATGCGTCTCCTCAGTAGGTCTGC*NNNNNN*GACTGATCCTAGTGCAATGCGTCTGAGCCTGAGCTACAGCGATATAGCCTGGATTGTGAGCGTATTTGCTGTCAGAACCTCAGCTCATCATGTATGATGCTGTACCATCCTGCGATTTA, where *TAATACGACTCACTATA* is the T7 promoter; the A nucleotide immediately adjacent to the promoter sequence is the TSS; *NNNNNN* is a barcode sequence without A’s to distinguish different spike-ins in the same sequencing round (five different spike-ins in total). These spike-ins were generated by in vitro transcription with different ratios of m^6^Am/Am cap analog to achieve a m^6^Am stoichiometry ranging from 0% to 100%. No internal m^6^A modification was made. These RNA spike-ins were added to the RNA samples before CROWN-seq library preparation. In total, we generated three libraries with spike-ins. Therefore, we obtained 15 data points for each A nucleotide position (5 different barcodes × 3 replicates). All A positions analyzed in this study have a coverage >100 reads. Detailed data can be found in Table S3.

### 2.4. 5′ Isoform-Specific m^6^A Identification and Quantification of CROWN-Seq Data

We developed a customized computational pipeline to quantify m^6^A stoichiometry at the resolution of individual transcription start sites (TSSs). The CROWN-seq reads were first mapped as previously described as the mapping pipeline as previously described [14]. The resulting CROWN-seq alignments were then processed read-by-read to examine both the genomic coordinate of the transcription start site (i.e., the first nucleotide in a read) and the conversion state of every nucleotide mapped to an A nucleotide in the reference genome. In particular, when the A nucleotide is sequenced as G in the read, it indicates the adenosine is not methylated; while if the A nucleotide is sequenced as A, it indicates the adenosine might be m^6^A modified. Pysam (v0.19) [19] was used to perform BAM file processing. BAM file-related computation was performed on an Ubuntu 20.04.3 LTS environment. After iterating the BAM file, a table was generated, which contains the A or G reads of all the genomic A positions and their associated TSSs. This table was then processed for the identification and quantification of m^6^A sites as well as TSS-m^6^A-site pairs.

To call TSS-m^6^A-site pairs, we first identified the m^6^A sites. In this study, we focused on identifying high-confidence m^6^A sites. In previous modification studies, a modified site might be determined by ≥20 reads and ≥0.1 stoichiometry [14,20,21]. However, in this study, we used a stricter filter to avoid false positives and ensure quantification accuracy: a high-confidence m^6^A site must be (1) located in a DRACH motif; (2) have ≥50 reads and ≥0.2 stoichiometry in at least one 5′ isoform.

This filter is related to the non-conversion background, which is ~1.5% to 4% in different positions in a read. As estimated by the binomial distribution, the non-conversion background might influence m^6^A identification when the background non-conversion rate is slightly higher (e.g., 5%) than our expectation, or when the sequencing depth is low (e.g., 20 reads) (Figure S1). Therefore, we used a high coverage (≥50 reads) and high stoichiometry cutoff (≥0.2) to ensure both accuracy and precision.

After m^6^A site identification, we then quantify the stoichiometries in the corresponding TSS-m^6^A-site pairs. During this step, we only considered the TSS-m^6^A-site pairs that were covered by ≥50 reads. To increase sequencing depth for each cell type, we merged technical/biological replicates.

### 2.5. TSS and 3′ Exon End Annotation of TSS-m^6^A-Site Pairs

We directly calculated the distance between a TSS and an m^6^A site based on their relative position in a read. We did not use genomic coordinates of the TSS and the m^6^A sites directly because splicing might occur at the 5′ end.

To investigate the joint effects of transcript architecture, we annotated each m^6^A site with its distance from the 3′ end of the first exon. To do so, we first examined the splicing events in each read. If splicing was observed, we used the distance between the m^6^A site and the splicing site. If no splicing event was found in the read, we then examined the closest annotated 3′ exon end in Gencode v45 annotations. To do so, we used the “closest” subprogram of BEDTools [22] to annotate the distance between the TSS and all possible 3′ exon ends. A customized script was used to rank all the distances and find the closest 3′ exon end.

The related codes can be found on GitHub: https://github.com/jhfoxliu/TSS_m6A (accessed on 12 March 2026).

### 2.6. Linear Fixed-Effects Model

To quantify the impact of 5′ proximity on m^6^A stoichiometry, while controlling for site-specific variability, we employed a linear fixed-effects regression model. Analysis was performed using the Python package “Statsmodels” [23]. TSS-m^6^A-site pairs were first categorized into 5-nucleotide (nt) bins based on their distance from the 5′ end. We then modeled m^6^A levels using the formula: m^6^A level ~ C(5′ distance bin) + C(m^6^A site).

In this model, the m^6^A site was treated as a fixed effect to account for site-specific methylation efficiencies and local sequence contexts. The intercept represents the baseline methylation level of the reference bin (1–5 nt), while the coefficients for subsequent bins represent the mean deviation from this baseline. The model’s adjusted R^2^ was used to assess the proportion of variance explained by the combination of spatial positioning and site-specific factors. The detailed model output can be found in Table S1.

### 2.7. Motif Analysis

Motif analysis was performed with Weblogo 3.7.12 [24]. The 2 nt flanking region of the m^6^A site was used as input. Probability mode (--units probability) was used to generate the frequency logo.

### 2.8. Quantification and Statistical Analysis

Python (3.13.7) and its related packages were used to perform quantification and statistical analysis. Statistical analysis was performed with Numpy (v1.23.5), pandas (v1.5.2), Scipy (v1.9.3), and statsmodels (v0.14.2). Figures were generated with Matplotlib (v3.6.2) and Seaborn (v0.12.1). *p*-value significance levels: * (<0.05), ** (<0.01), *** (<0.001), **** (<0.0001).

## 3. Results

### 3.1. m^6^A and m^6^Am Are Easily Confused with Each Other in Non-5′ Specific Analysis

**Figure 1:**
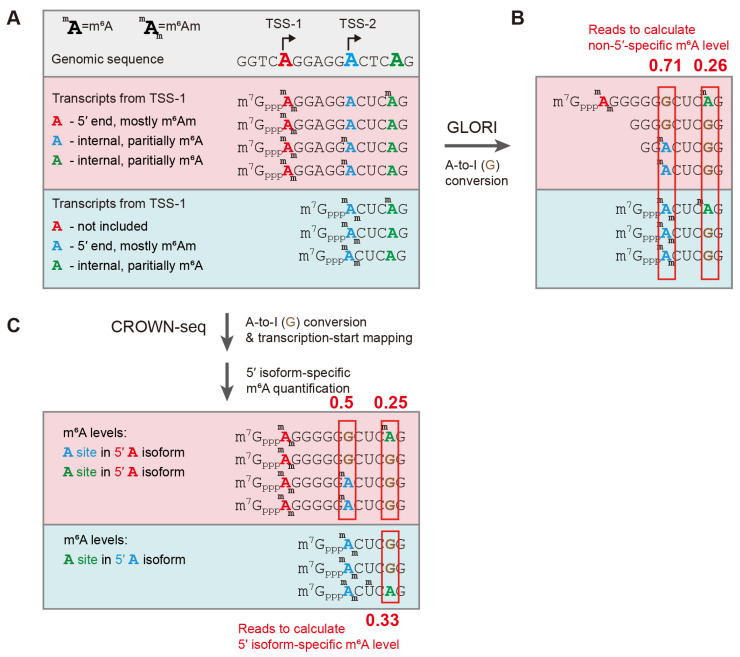
CROWN-seq provides accurate quantification of cap-proximal m^6^A sites: (**A**) The complex sequence architecture at the 5′ terminus, where a gene may utilize multiple genomic adenosines differently: a proximal site used exclusively as a transcription start site (TSS) (red), a middle site functioning as either a TSS or an internal site depending on isoform choice (blue), and a distal site used purely as an internal position (green). (**B**) Conventional methods like GLORI aggregate these signals, leading to inaccurate stoichiometry at the middle site due to the conflation of m^6^A and m^6^Am. Furthermore, for the distal site, non-5′-specific analysis fails to detect potential isoform-specific methylation dynamics. The way to calculate the methylation level and the results are indicated in red. (**C**) m^6^A quantification at 5′ isoform resolution using CROWN-seq. CROWN-seq resolves cap-proximal quantification issues by selecting and labeling RNA fragments by their m^7^G caps following GLORI chemistry conversion. This approach physically separates m^6^Am signals from internal sites, enabling the calculation of m^6^A stoichiometry for each specific 5′ isoform.

We sought to quantify the stoichiometry of m^6^A sites in mRNA 5′ UTRs. A major problem with previous studies of m^6^A in 5′ UTRs is the presence of m^6^Am, a modified nucleotide that is found exclusively at the first nucleotide position of RNA Pol II transcripts (Figure 1A). In previous antibody-based methods, like MeRIP-seq, the m^6^A antibodies also bind m^6^Am, causing peak enrichments in mRNA 5′ regions. In many studies, 5′ peaks are misattributed to m^6^A, when they are instead much more likely to be m^6^Am. We partially addressed this by our discovery of the m^6^Am-forming enzyme, PCIF1 [25], and subsequent mapping of m^6^A in *PCIF1* knockout cells [14]. Although this study showed that most 5′-associated peaks were due to m^6^Am, it was clear that m^6^A is located in 5′ regions of RNA Pol II transcripts. However, MeRIP-seq does not provide quantitative measurements of m^6^A.

More recently, the GLORI method for mapping m^6^A was developed for quantitative measurements of m^6^A [12]. In this method, sodium nitrite is used to deaminate adenosines to inosine, while m^6^A is resistant to sodium nitrite-mediated deamination. During sequencing, inosines are read as G, while m^6^A sites are read as A. The fraction of reads that contain A rather than G at any individual site indicates the stoichiometry of m^6^A at any position (Figure 1B). However, certain limitations may affect the ability to detect m^6^A in 5′ regions using GLORI. First, the original GLORI dataset showed low coverage at 5′ ends, which was related to the fragmentation that occurs in the GLORI protocol. Second, the small fragments obtained using GLORI could not be definitively assigned to specific 5′ isoforms. Thus, the stoichiometry of m^6^A sites near the 5′ ends of mRNA reflects an average stoichiometry across all the 5′ isoforms for any gene.

We first assessed the existing GLORI dataset to determine if 5′ m^6^A sites could be detected. We examined the previously published GLORI dataset derived from HEK293T cells. Among the 170,240 high-confidence sites identified by GLORI [12], approximately 6016 are located within 100 nucleotides downstream of a TSS. Since m^6^A sites have previously been confused with m^6^Am sites, we compared the locations of these sites with transcription-start nucleotides identified with ReCappable-seq [26], a method for mapping the precise position of the first nucleotide in mRNAs. Notably, about half of the m^6^A sites in 5′ regions (n = 3246) directly overlapped with a TSS, raising the possibility that a considerable fraction of m^6^A sites mapped to 5′ ends in GLORI in fact are m^6^Am.

To address this problem, we decided to use our recent CROWN-seq dataset, which was originally developed to quantify m^6^Am stoichiometries in HEK293T mRNA [14]. CROWN-seq involves selective labeling of the 5′ ends of mRNA using a decapping and recapping step, which ensures that true capped 5′ ends of RNAs are recovered for analysis. These 5′ fragments are subjected to the GLORI chemistry, which we showed can convert 2′-*O*-methyl adenosines at the first nucleotide position of mRNAs into 2′-*O*-methyl inosines, but leaves m^6^Am unmodified. Similar to GLORI measurements of m^6^A, the stoichiometry of m^6^Am can be assessed by measuring the fraction of reads that remain as A (Figure 1C).

**Figure 2:**
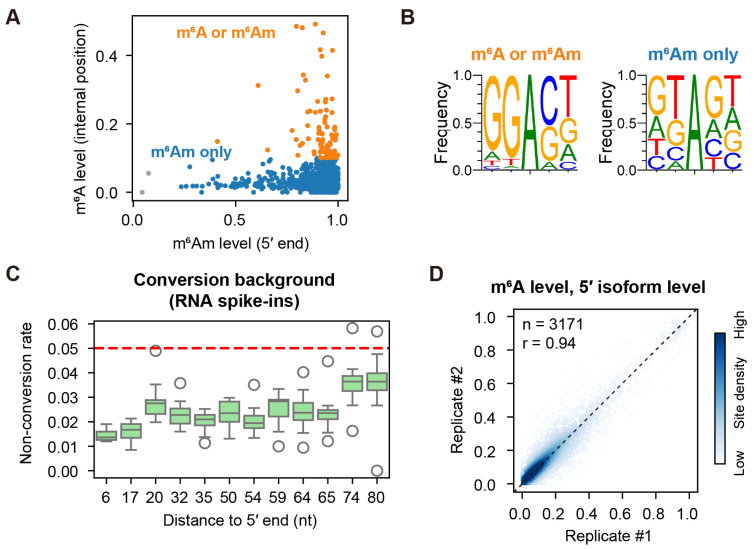
CROWN-seq provides accurate cap-proximal m^6^A quantification: (**A**) Global mischaracterization of m^6^Am as m^6^A in non-5′-specific datasets. Comparison of m^6^Am levels at 5′ ends (X-axis) versus non-5′-specific m^6^A stoichiometry for the same genomic adenosine when transcribed as an internal site (Y-axis). The methylation levels are obtained from CROWN-seq. Data points highlighted in orange represent sites with internal m^6^A levels exceeding 0.1, identifying a subset of adenosines with dual modification identities. The CROWN-seq technical replicate #1 was used. (**B**) Corresponding to (**A**), shown are the motif logos of the sites that are modified as both m^6^Am and m^6^A, or the sites merely modified as m^6^Am. (**C**) The non-conversion background in CROWN-seq estimated by RNA spike-ins. In this analysis, we plotted the non-conversion rates of adenosines in the RNA spike-in sequence between position 6th and 80th. (**D**) High reproducibility of isoform-specific m^6^A quantification by CROWN-seq. Scatter plot demonstrating robust correlation of m^6^A stoichiometry in TSS-m^6^A-site pairs between technical replicates.

We then further explored our CROWN-seq dataset, but instead focused on m^6^A sites that were internally located in each read. In this analysis, we examined 2231 of the 3246 m^6^A sites sequenced by both GLORI and CROWN-seq, which have high coverage in CROWN-seq (i.e., at least 50 reads) in two technical replicates. We observed that the majority of these sites are predominantly m^6^Am with high stoichiometry (typically >0.9), indicating that m^6^Am sites can easily be mischaracterized in GLORI datasets (Figure 2A). Remarkably, we identified 92 sites that exhibit both m^6^A and m^6^Am signatures, i.e., m^6^A in one transcript isoform and m^6^Am in another (Figure 2A; Table S2). Motif analysis showed the sites being modified as m^6^A and m^6^Am are in classic DRACH motifs, while the sites preferentially modified as m^6^Am are mostly non-DRACH motifs (Figure 2B). Therefore, in the traditional GLORI-based m^6^A analysis, m^6^A and m^6^Am would not be distinguished from each other.

### 3.2. CROWN-Seq Enables m^6^A Stoichiometry Quantification at 5′ Isoform Resolution

A primary advantage of CROWN-seq is its ability to simultaneously capture the precise transcription-start nucleotide, which thus reveals the exact 5′ isoform and internal m^6^A stoichiometry. This unique feature allows m^6^A modifications to be attributed to specific 5′ transcript isoforms, providing a resolution that is often lost in non-5′-specific sequencing. Consequently, CROWN-seq uniquely reveals m^6^A stoichiometry, which may be different depending on the specific 5′ isoform.

While we previously demonstrated that CROWN-seq achieves high precision in quantifying m^6^Am stoichiometry at the +1 position [14], it was essential to verify the efficiency of sodium nitrite-mediated deamination within internal read regions. To evaluate the background non-conversion rate, we analyzed the synthetic RNA spike-ins utilized in our prior study [14], which contain unmodified internal adenosines and a modified m^6^Am at the first nucleotide. Our analysis revealed high conversion efficiency across internal adenosine residues: the background non-conversion rate at the 2nd and 17th nucleotide positions was approximately 1.5%, rising slightly to ~2.5% between the 20th and 65th nucleotides (Figure 2C, Table S3). At the 74th and 80th positions, the background rate reached approximately 3–4% (Figure 2C, Table S3). These results establish a low technical noise floor for internal m^6^A detection.

To further validate the technical robustness of CROWN-seq, we compared m^6^A site identification across two independent HEK293T technical replicates. We applied a high-stringency filter (50 reads and ≥0.2 stoichiometry in at least one 5′ isoform, see Figure S1) to identify internal m^6^A sites across different 5′ isoforms (Figure S3A). This led to the identification of 650 DRACH-context m^6^A sites across 2861 transcription start sites (TSSs), resulting in 2982 high-confidence TSS-m^6^A-site pairs. We observed a high degree of correlation in m^6^A stoichiometry across the two replicates based on non-conversion rates (Pearson’s r = 0.94; Figure 2D). Notably, 274 of the identified m^6^A sites had not been previously reported by GLORI [12], including sites within essential genes such as *MCM7*, *ATG5*, and *PTTG1* (Table S4). Collectively, these data demonstrate that CROWN-seq is a robust and reproducible method for the identification and quantification of m^6^A at 5′ isoform resolution.

### 3.3. An Inhibitory Effect of m^6^A Deposition Around the Cap-Proximal Region

**Figure 3:**
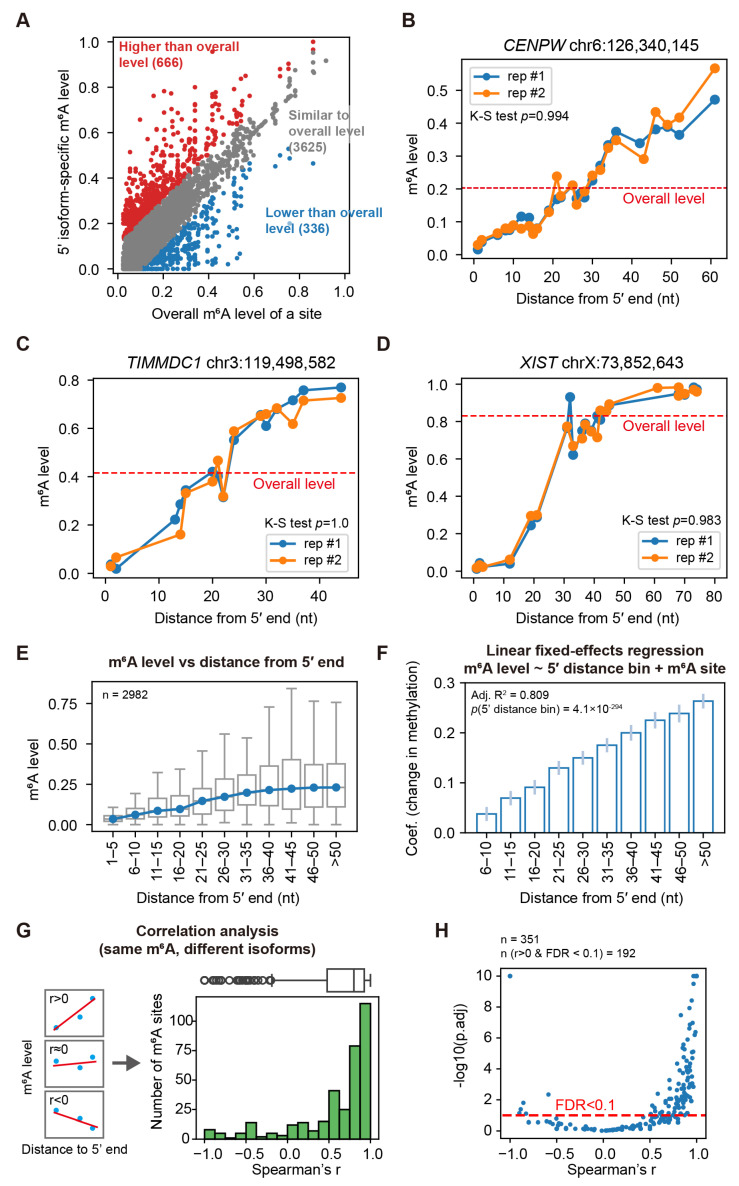
m^6^A deposition is inhibited in the cap-proximal region: (**A**) Heterogeneity of m^6^A stoichiometry across distinct 5′ isoforms. Comparison of overall m^6^A levels versus 5′ isoform-specific m^6^A stoichiometry derived from CROWN-seq. Each data point represents a unique TSS-m^6^A-site pair. Pairs exhibiting markedly lower methylation levels (stoichiometry < 0.1) relative to the overall stoichiometry of a site are indicated in blue, while those with elevated methylation levels are highlighted in red. (**B**–**D**) Representative loci demonstrating cap-proximal m^6^A inhibition. Examples of m^6^A sites from the CENPW, TIMMDC1, and XIST transcripts show a marked reduction in stoichiometry when positioned near the 5′ terminus. Overall methylation levels of the site are represented by red dashed lines, with isoform-specific stoichiometry from two technical replicates displayed for comparison. Kolmogorov–Smirnov (K-S) tests were performed to examine whether the distribution of m^6^A levels between replicates was different or not. (**E**) Transcriptome-wide positive correlation between m^6^A stoichiometry and cap distance. Grouped analysis shows that m^6^A levels increase as the distance from the m^7^G cap increases. Median stoichiometry values for each distance interval are plotted in blue and connected to highlight the distance-dependent inhibitory effect. (**F**) Corresponding to (**E**), distance from the 5′ cap is positively associated with m^6^A stoichiometry. Plotted are the regression coefficients (Y-axis) for each 5′ distance bin (X-axis) derived from a linear fixed-effects model (*m^6^A level ~ 5′ distance bin + m^6^A site*). This model considers both the effect of the 5′ distance and the site-specific m^6^A deposition efficiency. The model achieves a high adjusted R^2^ (0.802), indicating that the spatial position relative to the TSS, combined with site-specific factors, explains a substantial proportion of the variance in methylation levels. A higher coefficient indicates a more positive contribution to the m^6^A level. Error bars represent 95% confidence intervals (CIs). *p*-values of the coefficient are shown in asterisks. (**G**) Site-specific correlation analysis confirms widespread inhibition. Distribution of Spearman correlation coefficients calculated between m^6^A stoichiometry and distance from the 5′ cap for 351 individual m^6^A sites. The predominant shift toward positive coefficients supports the model that m^6^A deposition is systematically repressed in the cap-proximal region across the transcriptome. (**H**) Corresponding to (**G**), among the 351 Spearman’s correlation tests, 192 tests were positive and significant after multiple testing (FDR, Benjamini–Hochberg method). In this plot, FDR values smaller than 1 × 10^−10^ were shown as 1 × 10^−10^.

We next investigated whether m^6^A stoichiometry at a single site varies across different 5′ isoforms. To address this, we compared the non-5′-specific (overall) m^6^A stoichiometry of a site with 5′ isoform-specific stoichiometry using HEK293T CROWN-seq data. While many sites exhibited consistent methylation levels across isoforms, a significant proportion showed substantially higher stoichiometry (>0.1) in specific 5′ isoforms compared to the overall estimation (Figure 3A). Conversely, we identified instances where isoform-specific methylation was markedly lower (<0.1) than overall stoichiometry (Figure 3A), indicating that m^6^A stoichiometry is highly specific to the 5′ isoform context.

Our analysis revealed that this isoform-specific variation is strongly dictated by the spatial position of the m^6^A site relative to the transcription start site. For example, at the *CENPW* locus (chr6:126,340,145), a single m^6^A site is associated with over 25 distinct 5′ isoforms. We observed a strong negative correlation between methylation and proximity to the 5′ end: isoforms where the site was located within the first 10 nucleotides exhibited methylation levels below 0.1, significantly lower than the overall level of ~0.2 (Figure 3B and Figure S2). However, in isoforms where the TSS was positioned >20 nt upstream of the same site, stoichiometry increased to 0.3–0.5 (Figure 3B and Figure S2). The Kolmogorov–Smirnov test shows this methylation pattern is consistent between the two replicates. We observed similar distance-dependent inhibition at other loci, including *TIMMDC1* (chr3:119,498,582; Figure 3C) and the long non-coding RNA XIST (chrX:73,852,643; Figure 3D).

To determine if this “cap-proximal exclusion zone” is a transcriptome-wide phenomenon, we grouped TSS-m^6^A-site pairs by their distance from the TSS. To ensure high quantitative accuracy, we merged reads from two HEK293T technical replicates and selected high-confidence sites (at least 50 reads and ≥0.2 stoichiometry in at least one 5′ isoform; see Section 2). This yielded 2982 TSS-m^6^A-site pairs originating from 650 DRACH sites. By binning these pairs by TSS distance, we observed that m^6^A stoichiometry is positively correlated with the distance from the 5′ end (Figure 3E). This pattern is found in all subtypes of DRACH motifs (Figure S3B,C).

To account for the inherent variability in methylation efficiency between different DRACH sites, we employed a linear fixed-effects regression model. This model incorporated both the categorical distance bins and the specific m^6^A site as factors (see Section 2). The regression confirmed that distance to the 5′ end remains a significant positive predictor of methylation levels, even when controlling for site-specific baseline efficiencies (Figure 3F; Table S1).

Importantly, sampling bias might contribute to an artifact in our statistics above, because different m^6^A sites are associated with varying numbers of TSS-m^6^A-site pairs. To ensure our observations were consistent across individual loci, we performed a site-specific correlation analysis. To do so, we calculated Spearman’s correlation coefficients between the distance from the 5′ end and m^6^A stoichiometry for 351 m^6^A sites associated with at least three alternative TSSs. Overall, we found that the majority of the m^6^A sites exhibit a higher methylation when it is located further away from the 5′ end (Figure 3G). Notably, 192 of these sites (54%) exhibited significantly positive correlations (r > 0, FDR < 0.1; Figure 3H).

Collectively, these transcriptome-wide analyses in HEK293T cells demonstrate a pervasive cap-proximal exclusion zone that governs m^6^A deposition in the first 30 to 40 nt region.

### 3.4. Cap-Proximal m^6^A Deposition Inhibition Is Seen in Multiple Human Cell Types

To determine whether the cap-proximal m^6^A inhibition effect is a general regulatory principle rather than a phenomenon unique to HEK293T cells, we extended our analysis to eight additional human cell lines from our previous CROWN-seq study [14]: K562, A549, CCD841 CoN, HCT116, HT29, HepG2, Huh7, and Jurkat E6.1.

Consistent with our initial HEK293T analysis, we applied a stringent filtering pipeline to identify high-confidence TSS-m^6^A-site pairs. Biological and technical replicates were merged, and sites were required to possess at least one 5′ isoform with a minimum coverage of 50 reads and a methylation stoichiometry of ≥0.2. Across these diverse cellular contexts, we identified a total of 1346 m^6^A sites within canonical DRACH motifs, representing 6296 high-confidence TSS-m^6^A-site pairs. This dataset is available via Zenodo (10.5281/zenodo.19546831).

We subsequently modeled the relationship between TSS distance and m^6^A levels across all evaluation pairs in these additional cell lines. Remarkably, we observed a robust and consistent correlation between distance from the 5′ cap and methylation levels across every cell line tested (Figure S4). These results demonstrate that cap-proximal m^6^A inhibition is a pervasive feature of the human epitranscriptome, suggesting a fundamentally conserved mechanism governing m^6^A deposition at the 5′ terminus of mRNAs.

### 3.5. TSS Usage Influences m^6^A Stoichiometry in the 5′ Region

The observed heterogeneity in m^6^A stoichiometry at the isoform level suggests that the overall methylation level that is calculated at a specific site is a composite value, determined by the weighted average of the methylation stoichiometry in each 5′ isoform. We hypothesized that differential TSS usage across cell types could therefore markedly impact the overall methylation stoichiometry of specific m^6^A sites, especially in the 5′ UTR.

**Figure 4:**
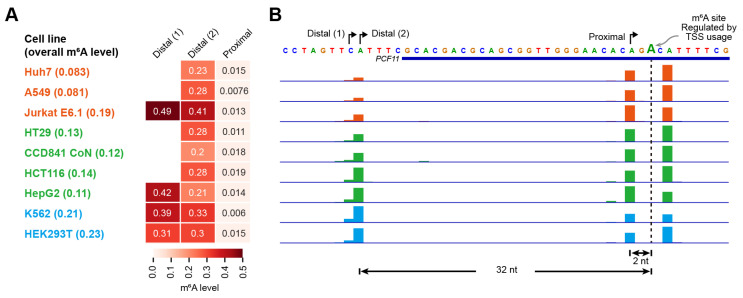
TSS usage determines overall m^6^A stoichiometry in *PCF11* transcripts: (**A**) Heatmap showing the methylation levels of the site chr11:83,157,157 in different isoforms across nine human cell lines. The overall methylation levels are also shown in brackets. The cell lines in orange preferentially use the proximal TSS; the cell lines in green tend to use both distal and proximal TSSs; the cell lines in blue preferentially use the distal TSS(s). Detailed coverages and methylation levels are available in Table S5. (**B**) Relative TSS usages of site chr11:83,157,157 across different cell lines.

To test this idea, we examined genes with clear cell type-specific alternative 5′ isoform usage and that contain m^6^A sites near the TSS. A representative example is the m^6^A site at chr11:83,157,157 within the *PCF11* gene. Based on CROWN-seq, we observed that the overall calculated stoichiometry at this site varies significantly across cell lines: in Huh7 and A549 cells, the overall stoichiometry is relatively low (~0.08), whereas in HEK293T and K562 cells, it increases more than two-fold to 0.21–0.23 (Figure 4A).

When examining the 5′ isoform usage, it was clear that this variation is directly attributable to a switching in the usage of specific 5′ isoforms. In one 5′ isoform (the “proximal” isoform), the m^6^A site is only two nucleotides away from the TSS, and exhibits almost no detectable methylation (Figure 4A). In contrast, other 5′ isoforms (“distal” isoforms) of *PCF11* have their TSS ~30 nt from the m^6^A site, resulting in 0.2–0.49 m^6^A stoichiometry in these isoforms (Figure 4B). Importantly, Huh7 and A549 cells preferentially utilize the proximal 5′ isoform, leading to an abundance of unmethylated transcripts and an overall low level of methylation at this site. In contrast, HEK293T and K562 cells preferentially express distal isoforms, thereby leading to an overall increase in m^6^A stoichiometry at this site (Figure 4A,B). Interestingly, in Jurkat E6.1 cells, while the proximal TSS is still favored, the exceptionally high methylation frequency of the few distal-TSS isoforms provides a compensatory effect, maintaining an intermediate overall methylation level (Figure 4A,B).

Taken together, the case of *PCF11* illustrates that m^6^A stoichiometry can be regulated by differential TSS usage, providing a mechanism for cells to tune the stoichiometry at specific m^6^A sites by switching the usage of specific 5′ isoforms.

### 3.6. Methylation Pattern in the First Exon Is Primarily Regulated by the Cap-Proximal Exclusion Zone

**Figure 5:**
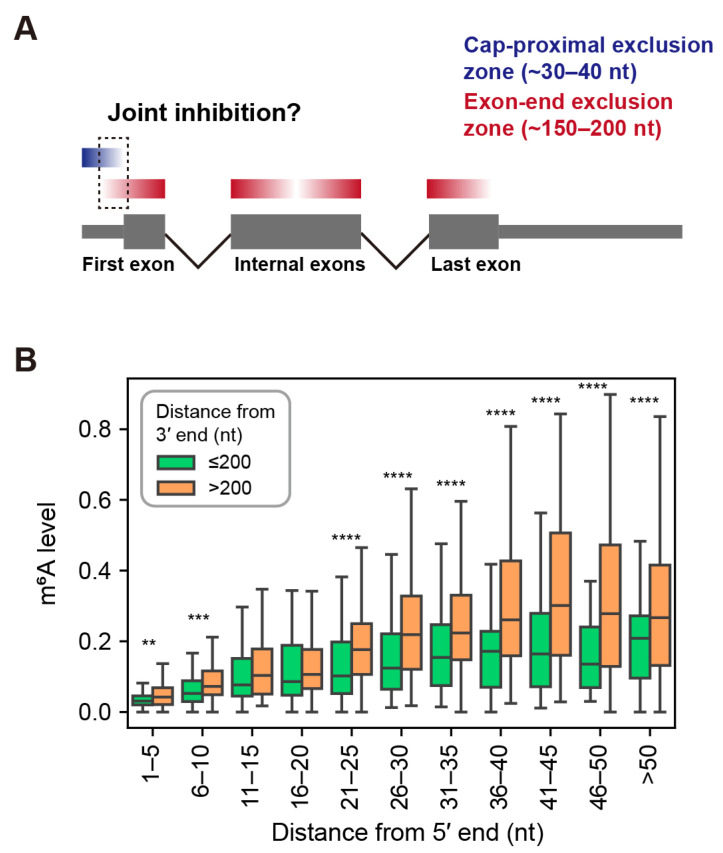
A joint inhibition effect of the cap-proximal exclusion zone and 3′ exon-end exclusion zone: (**A**) Spatial intersection of m^6^A inhibitory mechanisms. This schematic illustrates the potential overlap between the cap-proximal exclusion zone (blue) and the exon-end exclusion zone (red). (**B**) The m^6^A sites located in the 3′ exon-end exclusion zone undergo joint inhibition. Shown are stoichiometries of two groups of m^6^A sites, which are located ≤200 nt or >200 nt from the 3′ exon end. 1296 and 1684 TSS-m^6^A-site pairs were analyzed in these two groups, respectively. *p*-values, non-parametric Mann–Whitney U test to compare the ≤200 nt and >200 nt populations in each bin. Detailed *p*-values can be found in Table S1. *p*-value significance levels: ** (<0.01), *** (<0.001), **** (<0.0001).

We then asked whether m^6^A deposition is regulated by additional mechanisms beyond the cap-proximal exclusion zone. In the case of internal exons, suppression of m^6^A near exon–exon junctions occurs over ~150–200 nucleotides from the exon ends, and is thought to be driven by the exon junction complex [7,8,9,10]. In the last exon, only one end of the exon is bound by the splicing machinery, leading to an increase in m^6^A stoichiometry around the stop codons [7,8,9,10]. Notably, the first exon also only has one end bound by the splicing machinery. Since the median length of human 5′ UTRs is approximately 218 nucleotides [27], the first exon might be simultaneously regulated by both 5′ cap-proximal and 3′ exon-end exclusion zones (Figure 5A).

To test this idea, we first assessed the range of cap-proximal exclusion zone without the interference of exon-end suppression. We therefore established a control group of the m^6^A sites. These m^6^A sites are located >200 nt from the 3′ end of the first exon and therefore are unlikely to be influenced by the exon-end exclusion zone. We found that the cap-proximal exclusion zone of these sites is around 30 to 40 nt long, consistent with our findings above (Figure 5B).

We then asked whether the m^6^A sites close to the 3′ exon end exhibit joint inhibition by both the cap-proximal and the exon-end exclusion zones. We therefore selected the m^6^A sites ≤200 nt from the 3′ end of the first exon, presumably located within the 3′ exon-end exclusion zone. We found that these m^6^A sites have increased stoichiometry as the distance from the 5′ end increases, indicating the cap-proximal inhibition. However, compared with the control group, the stoichiometries of these m^6^A sites increased significantly more slowly as the distance from the cap increased (Figure 5B). For instance, for sites located >200 nt from the 3′ end, the median stoichiometry reached 0.3 when they are 41–45 nt away from the cap. In contrast, the sites ≤200 nt from the 3′ end exhibit a median stoichiometry of 0.16 in this region, which is ~47% lower. Therefore, these m^6^A sites undergo a joint inhibition due to the overlapping of the cap-proximal and the 3′ exon-end exclusion zones.

## 4. Discussion

Understanding the regulation of m^6^A deposition is necessary to determine how m^6^A might be regulated in different cellular contexts. Recently, it was proposed that m^6^A deposition is regulated primarily by an inhibitory effect of the splicing machinery, specifically the EJC, at exon–exon junctions [7,8,9,10]. Although this model effectively explains m^6^A levels within internal exons, this model does not address how m^6^A stoichiometry is determined in 5′ regions of RNA. Understanding how m^6^A levels in 5′ exons are regulated is further complicated due to the complex and often inaccurate mapping of mRNA 5′ ends [14]. In this study, we used a nucleotide-resolution map of transcription-start sites, along with a chemical method for exact quantification of m^6^A, and found that the stoichiometry of the cap-proximal m^6^A sites is repressed around the cap-proximal region within the first 30 to 40 nt of the TSS. We found that m^6^A stoichiometry is thus regulated by the choice of the transcription-start nucleotide in different transcript isoforms. We also found that m^6^A levels can be jointly suppressed by both the cap-proximal and 3′ exclusion zones of the first exon. This finding indicates that m^6^A stoichiometry is highly repressed in short first exons <200 nt, while high stoichiometry m^6^A sites are detected in large first exons. Overall, our results show that m^6^A stoichiometry in 5′ regions is determined by distinct exclusion zones at the 5′ and 3′end of the first exon, and can be dynamically regulated by TSS usage.

Our study demonstrates that previous m^6^A studies related to the cap-proximal regions may have yielded inaccurate estimates of cap-proximal m^6^A stoichiometry. These previous studies relied on methods that did not distinguish between different 5′ isoforms. In this study, we re-analyzed our previous CROWN-seq datasets, which provide the stoichiometry of m^6^Am and internal m^6^A for each specific 5′ isoform. This allows, for the first time, accurate quantitative profiling of these cap-proximal m^6^A sites for each 5′ isoform. This analysis revealed that m^6^A stoichiometry can be highly different based on its distance from the TSS.

Our data suggest a new function for alternative TSSs: to regulate m^6^A stoichiometry. Alternative TSSs are known to regulate gene expression by changing the regulatory elements in the 5′ UTR region. Different RNA 5′ isoforms can exhibit variable 5′ regulatory elements, including upstream ORFs (uORFs), RNA-binding protein binding motifs, 5′ terminal oligopyrimidine motifs, and pyrimidine-rich translational elements [28]. In addition to these elements, TSS switching from adenosine to non-adenosine also leads to the loss of m^6^Am at the first nucleotide [14]. Our study shows that a ~10-nucleotide switching in TSS usage might lead to a significant change in m^6^A stoichiometry for an internal cap-proximal DRACH motif. Notably, TSS switching has been observed in diverse cellular contexts, such as different tissues and developmental stages, and is often attributed to different activities of different transcription factors that may use different spatially proximal promoters [29,30]. TSS switching can also be seen as a result of mutations in general transcription factors or RNA Pol II [28] or genetic mutations in the promoter region [31], or as a result of various environmental stimuli (e.g., hypoxia) or drug treatment [32].

The resulting change in m^6^A stoichiometry might significantly influence the binding of m^6^A readers to the cap-proximal region, for example, the binding of YTHDF1/2/3, YTHDC1, and other proteins [4,33]. These m^6^A readers could influence translation, RNA stability, RNA subcellular location, or other functions [4]. In addition, the gain or loss of m^6^A in the 5′ end could function as a regulator of RNA structure in the 5′ UTR since m^6^A tends to disrupt base pairing [34]. Therefore, our study reveals a new mechanism by which transcription-start site choice can influence gene expression, i.e., by altering m^6^A stoichiometry.

One potential model for reduced methylation near the TSS is that the RNA is too short to be a substrate for the METTL3/METTL14 writer complex. Indeed, a previous study showed that RNA substrates should be at least an 8–11 nt RNA strand for efficient m^6^A deposition [35]. Methylation rates achieved highest efficiency when the substrate was 13–14 nt long [35]. However, this is unlikely to fully explain the results here. We observed a gradual increase in m^6^A deposition within the first 30–40 nt from the TSS. Therefore, the cap-proximal exclusion zone cannot be simply explained by the size of the RNA substrate adjacent to the cap.

Here, we propose two different but related models to explain the 5′ cap proximal exclusion zone. First, similar to the inhibitory effect of the splicing machinery, the cap-binding complex (CBC) and its associated factors [36,37,38] might also prevent access of the METTL3/METTL14 writer complex to the 5′ end of mRNAs. The second model is that the recruitment of the m^6^A writer complex requires a specific transcriptional state, for example, a specific phosphorylation state of the C-terminal domain (CTD) of RNA Pol II [39]. The lack of such a signal during the very beginning of transcription might lead to a lag of m^6^A deposition. Future studies will reveal the precise mechanism of the suppressive effect of the 5′ cap proximal exclusion zone.

A limitation in our analysis is that CROWN-seq reads are generally short due to the highly fragmenting conditions of RNA deamination (i.e., 30–60 nt on average). In our dataset, many reads do not contain the entire first exon. As a result, the 3′ end of the exon needs to be inferred based on genomic annotations. This read length limitation also prevents us from investigating the exact cap-proximal exclusion zone for every m^6^A site. Future studies might use newer RNA deamination methods with less fragmentation [40] or full-length direct RNA sequencing of nascent RNA to reveal the diversity of m^6^A deposition around the 5′ ends.

## Data Availability

The comprehensive dataset of TSS-m^6^A-site pairs is available on Zenodo (10.5281/zenodo.19546831).

## Supplementary Materials

The following supporting information can be downloaded at https://www.mdpi.com/article/10.3390/genes17060653/s1. Figure S1. Evaluation of m6A call threshold by binomial distribution; Figure S2. Corresponding to Figure 3B; Figure S3. Corresponding to Figure 3E; Figure S4. Cap-proximal inhibition across other human cell lines. Table S1, summary of statistical analyses; Table S2, the A nucleotides transcribed as both m^6^A and m^6^Am; Table S3, the non-conversion background in RNA spike-in transcripts; Table S4, 5′ isoform-specific m^6^A stoichiometry in HEK293T cells; Table S5, methylation level and coverage of 5′ isoforms of *PCF11* loci chr11:83,157,157.

## Abbreviations

The following abbreviations are used in this manuscript:

m^6^A	*N*6-methyladenosine
m^6^Am	*N*6,2′-*O*-dimethyladenosine
TSS	Transcription start site
5′ UTR	5′ Untranslated region
ERCC	External RNA Controls Consortium
RNA Pol II	RNA Polymerase II
EJC	Exon junction complex
GLORI	Glyoxal and nitrite-mediated deamination of unmethylated adenosines
CROWN-seq	Conversion Resistance detection On Whole-transcriptomic transcription-start *N*6,2′-*O*-dimethyladenosine by sequencing
CTD	C-terminal domain
*CENPW*	Centromere protein W
*TIMMDC1*	Translocase of Inner Mitochondrial Membrane Domain Containing 1
*XIST*	X Inactive Specific Transcript
CBC	Cap-binding complex
uORF	Upstream open reading frame
FDR	False discovery rate
BH	Benjamini–Hochberg
PMF	Probability mass function
BAM	Binary alignment map
CI	Confidence interval
