# Supplementary material for: Transcription Start Site Choice Regulates m6A Stoichiometry in Cap-Proximal Regions
Source: Genes (Basel). 2026 May 31;17(6):653. doi: 10.3390/genes17060653 (PMC13298977; doi:10.3390/genes17060653)
Supplement: Supplementary file 1 [file genes-17-00653-s001.zip › Supplemental figures.pdf]

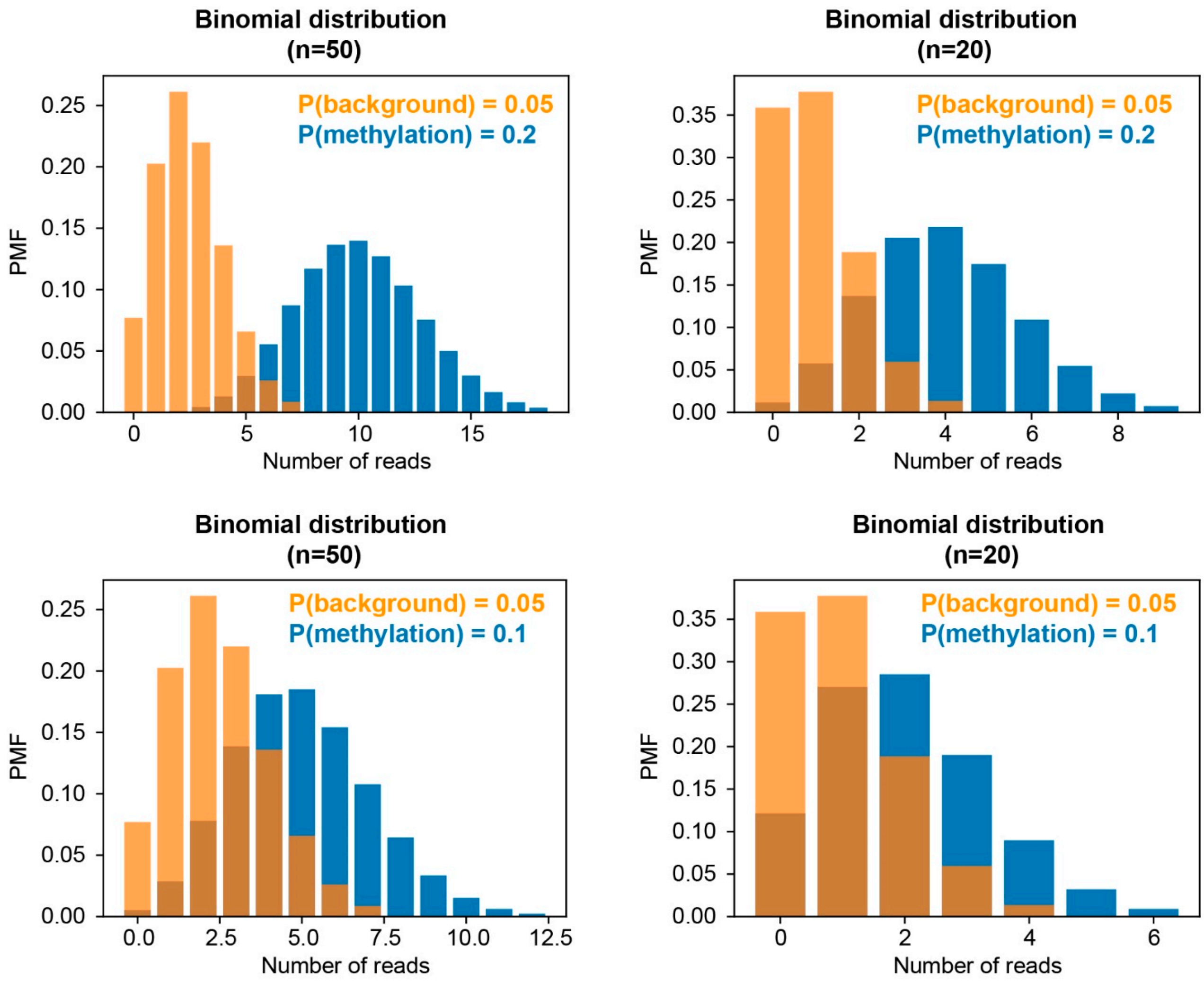

Figure S1. Evaluation of m^{6}A call threshold by binomial distribution.

The probability mass function (PMF) distribution of the number of false positives and the corresponding true positive or artificial stoichiometry level was estimated by a binomial distribution. In these estimations, the non-conversion rate background was set to 5% (in orange), which is slightly higher than the real situation of the CROWN-seq libraries. The probability of true positives (in blue) was set to 0.1 or 0.2. Sequencing depth (n) of 20 or 50 reads was simulated. As shown in the upper left panel, the threshold (≥50 reads and ≥0.2 stoichiometry) well separates the methylation signal from the background. Once a true positive site is determined, the 50 reads cutoff also provides enough precision in quantification, even considering the non-conversion background.

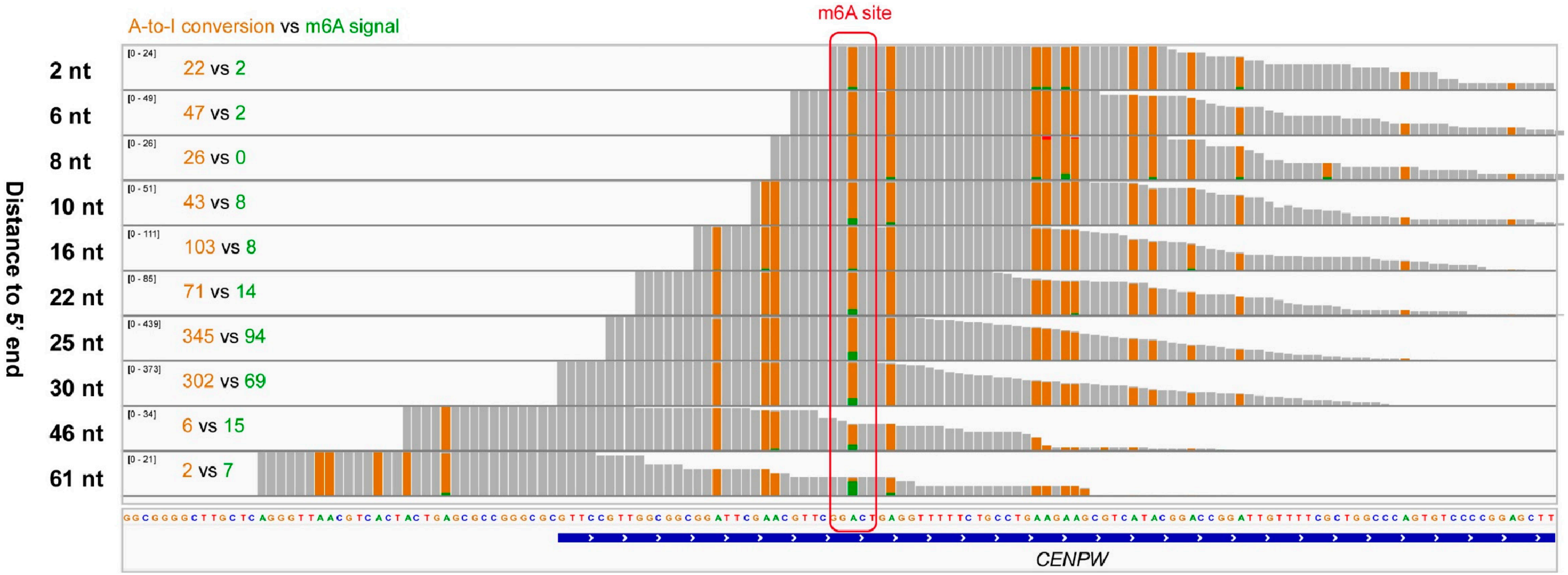

Figure S2. Corresponding to Figure 3B.

Shown is an IGV snapshot demonstrating the read coverages and methylation levels of specific 5′ isoforms of *CENPW*. In each row, shown are the reads from a specific 5′ isoform. The distances between the 5′ end and the m^{6}A site (highlighted in red) are shown on the left. The read number of A (A-to-I conversions) and m^{6}A are shown in brown and green, respectively.

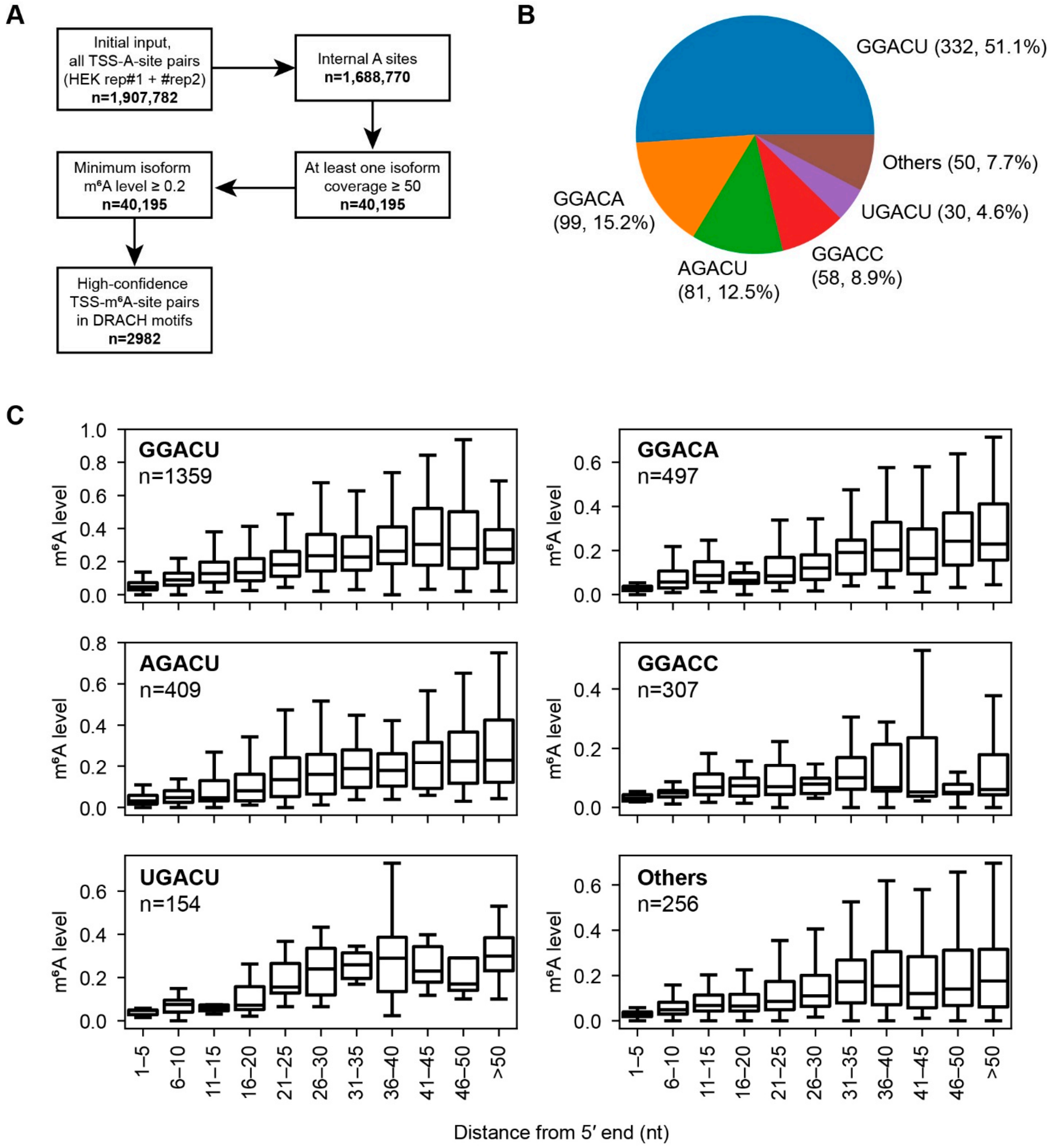

Figure S3. Corresponding to Figure 3E.

(A) The remaining number of TSS-m^{6}A-site pairs after every filtering step in the HEK293T dataset.

(B) The number and fraction of m^{6}A sites in different DRACH motifs in the HEK293T dataset.

(C) The cap-proximal exclusion zone is seen in multiple subtypes of DRCH motifs. The number of TSS-m^{6}A-site pairs is shown in each plot.

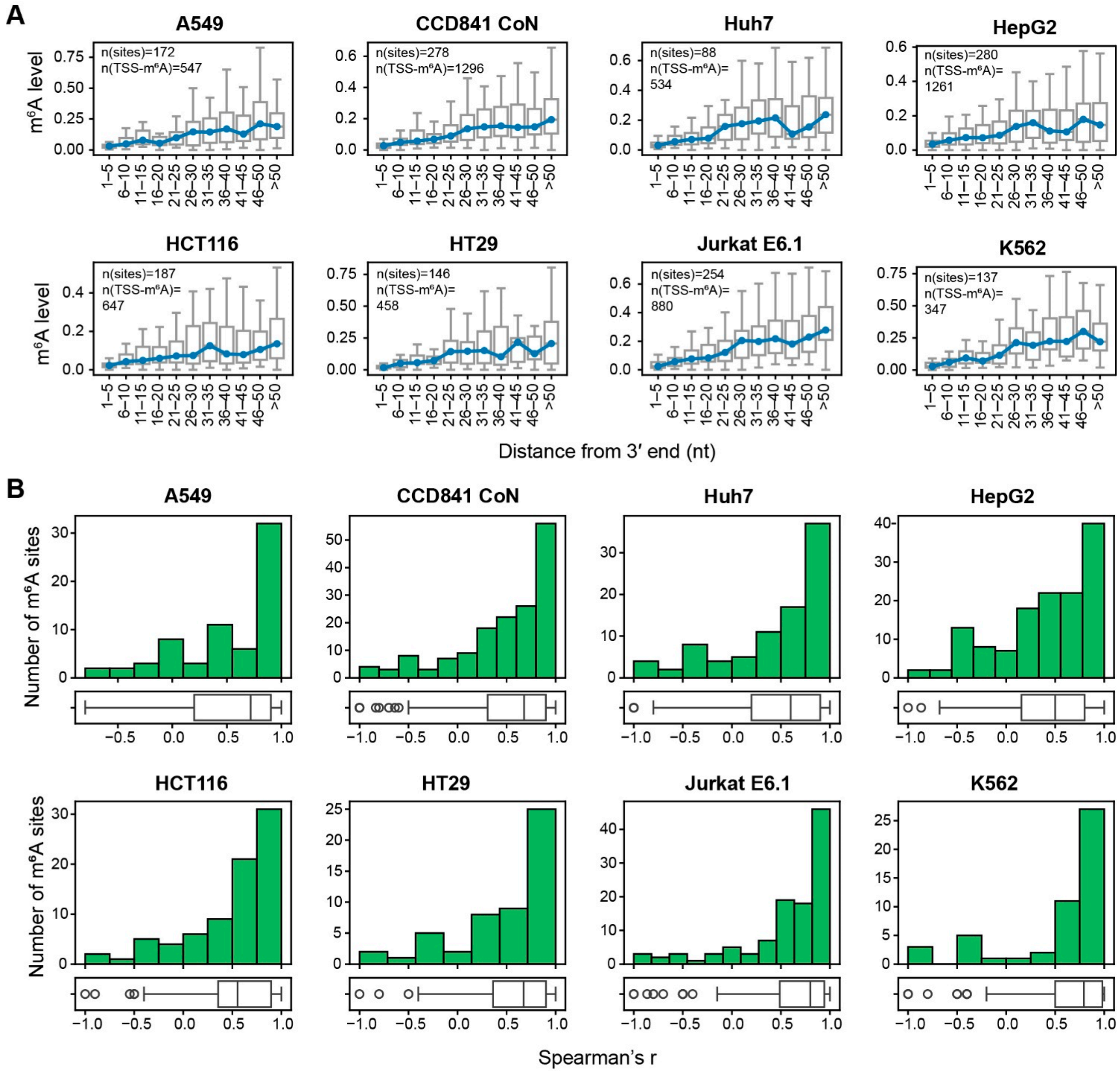

Figure S4. Cap-proximal inhibition across other human cell lines.

(A) Similar to **Figure 3E**, shown is the positive correlation between m^{6}A stoichiometry and 5′ distances across multiple cell lines.

(B) Similar to **Figure 3G**, shown are the site-specific analyses between m^{6}A stoichiometry and 5′ distances across multiple cell lines.
